# The challenges imposed by artificial intelligence: are we ready in medical education?

**DOI:** 10.1186/s12909-023-04660-z

**Published:** 2023-09-19

**Authors:** Samy A. Azer, Anthony P. S. Guerrero

**Affiliations:** 1 Department of Medical Education, College of Medicine, King Saud University, P O Box 2925, 11461 Riyadh, Saudi Arabia; 2 Department of Psychiatry, John A. Burns School of Medicine, University of Hawaii, Honolulu, USA

## Abstract

Artificial intelligence (AI) is the science and engineering of making intelligent machines. In medical education, the usefulness of AI and its applications is being explored in training, learning, simulation, curriculum, and developing new assessment tools. This editorial encourages authors to submit their research on AI concerning medical education to enrich our knowledge.

## Main text

In 2020, in the book titled "The Reality Game" Samuel Woolley raised crucial questions on how the next wave of technology will change our lives. Woolley discussed artificial intelligence (AI) and its applications in the field of education [1]. He raised several challenges that we will face as the field of AI grows. For example, Facebook Chief Executive Officer Mark Zuckerberg’s testimony before congressional lawmakers in Washington, D.C., demonstrated smart technology systems’ vulnerabilities – in allowing personal information to be misnhandled or in spreading politically oriented misinformation and propaganda, and potential strengths – in limiting the misuse of technology through deploying new tools to highlight the level of evidence behind each piece of information [2, 3]. Nowadays, machine-learning bots, such as Chat-GPT and alternatives – including Bard by Google, HuggingChat, Bing AI, Sparrow by DeepMind, YouChat, and others – can write Wikipedia articles, students’ assignments, novels, and book chapters, to name just a few. These new developments raise several questions about the suitability of current policies and regulations, copyright and ethical concerns, and what type of alternative assessment could be introduced to face these challenges in education and beyond.

The term Artificial Intelligence (AI) was first coined in 1956 [3]. It encompasses a variety of applications ranging from general ones, like learning and perception research, to highly specialised tasks; such as playing chess, writing poetry, driving a car, or diagnosing a disease. In 2007, John McCarthy, an American computer scientist and cognitive researcher who published widely in the field, defined AI as the science and engineering of making intelligent machines, including smart computer programs. It relates to using computers to understand human intelligence and the use of programs to execute and solve complex tasks [4].

With these abilities provided by AI in different applications and fields of our daily lives, current research on the use of AI in various specialties of medicine has addressed several interesting areas: medical images, nuclear medicine, cancer histopathology, cardiology, pediatric ophthalmology, and healthcare, drug discovery, and others [5]. The applications of AI are not limited to medicine; we need to benefit from the many applications AI provides in medical education and make required changes to our curricula, teaching and learning, and student assessment by introducing AI. Moreover, we must assess what policies and regulations should be introduced in light of such changes. In medical education, the uses of AI and its applications have been explored in several studies and a recent AMEE Guide [6]. Examples include, but are not limited to, potentially applying AI in surgical education and training [7], assessing readiness to changes in medical curricula and developing new assessment tools [8], reshaping the teaching of radiology [9], improving AI literacy in oral and dental education [10], and assessing surgical expertise [11].

The future physician will likely need to effectively and ethically incorporate AI in certain patient care tasks, like history-gathering, visual diagnosis, information synthesis, and decision-making. They will also need to productively collaborate with patients who use AI to assist with the self-management of acute and chronic conditions. On a larger systems level, medical professionals may employ AI in processes intended to improve healthcare operations, reduce error, decrease the load on hospitals, and ultimately ensure quality and sustainability. Finally, during both training and subsequent practice, the future physician may encounter AI in receiving feedback about learning and teamwork.

The new developments in AI may raise several questions concerning medical education, including:

- How could the changes produced by AI enforce changes in undergraduate and postgraduate medical and health curricula?
- How can we invest in social and learning programs that empower those disadvantaged in systems of power, including ethnic, religious, and other minority groups, particularly in education?
- How can AI assistance preserve the capacity of physicians to focus on tasks that only humans can perform, and, in turn, improve accessibility of healthcare, especially for vulnerable populations?
- What is the role of policymakers, tech companies, research institutes, and society in rebuilding the new functions of universities and medical and allied healthcare programs and training with the introduction of AI?
- What type of assessment should we use in higher education to replace MCQs, short answer, and essay questions?
- What are the emerging ethical and professional concerns we face with the new developments provided by AI systems in medical education.

In this Collection, we are looking for original research articles and systematic reviews on different aspects of medicine and allied health care education that can provide insight into improvement and challenges imposed by AI and explore how can we reshape our approaches based on current evidence.

## Data Availability

Not applicable.

## Abbreviations

AI: Artificial intelligence
AMEE: Association for Medical Education in Europe
